# Impact of a Combined High Cholesterol Diet and High Glucose Environment on Vasculature

**DOI:** 10.1371/journal.pone.0081485

**Published:** 2013-12-09

**Authors:** Zemin Wang, Yun Mao, Taixing Cui, Dongqi Tang, Xing Li Wang

**Affiliations:** 1 Shandong University Qilu Hospital Research Center for Cell Therapy, Key Laboratory of Cardiovascular Remodeling and Function Research, Qilu Hospital of Shandong University, Jinan, P. R. China; 2 Department of Cell Biology and Anatomy, South Carolina University, Columbus, South Carolina, United States of America; 3 Center for Stem Cell and Regenerative Medicine, The Second Hospital of Shandong University, Jinan, P.R. China; Thomas Jefferson University, United States of America

## Abstract

**Aims:**

Vascular complications are the leading cause of mortality and morbidity in patients with diabetes. However, proper animal models of diabetic vasculopathy that recapitulate the accelerated progression of vascular lesions in human are unavailable. In the present study, we developed a zebrafish model of diabetic vascular complications and the methodology for quantifying vascular lesion formation real-time in the living diabetic zebrafish.

**Methods and Results:**

Wild type zebrafish (AB) and transgenic zebrafish lines of *fli1:EGFP*, *lyz:EGFP, gata1:dsRed*, double transgenic zebrafish of *gata1:dsRed/fli1:EGFP* were exposed to high cholesterol diet and 3% glucose (HCD-HG) for 10 days. The zebrafish model with HCD-HG treatment was characterized by significantly increased tissue levels of insulin, glucagon, glucose, total triglyceride and cholesterol. Confocal microscopic analysis further revealed that the diabetic larvae developed clearly thickened endothelial layers, distinct perivascular lipid depositions, substantial accumulations of inflammatory cells in the injured vasculature, and a decreased velocity of blood flow. Moreover, the vascular abnormalities were improved by the treatment of pioglitazone and metformin.

**Conclusion:**

A combination of high cholesterol diet and high glucose exposure induces a rapid onset of vascular complications in zebrafish similar to the early atherosclerotic vascular injuries in mammalian diabetic models, suggesting that zebrafish may be used as a novel animal model for diabetic vasculopathy.

## Introduction

The latest statistics of National Institute of Health (NIH) indicate that diabetes is the seventh leading cause of death in the United States; vascular complications are the principal cause of morbidity and mortality in patients with type 1 and type 2 diabetes.[1] There appears to be no single animal model that encompasses all of these characteristics, but provides very similar characteristics in one or more aspects of type 2 diabetes in humans. On the other hand, the mechanisms underlying the accelerated progression of vascular disease seen in human diabetes are still not clear, and adequate animal models of diabetic vasculopathy remain to be further developed. [2].

Zebrafish are widely used to investigate developmental biology due to the advantages of relatively low cost, rapid lifecycle, and the optical transparency of zebrafish larvae. [3] A direct visualization of complex biological phenomena at the level of the entire zebrafish in a large scale is also achieved by novel imaging techniques. [4] Because of the revelation of zebrafish genome sequence as well as the development of gene targeting techniques in zebrafish, the generation of transgenic or gene knockout zebrafish is feasible.[5], [6], [7] Accordingly, zebrafish may be positioned as a unique and powerful animal model system to study dynamic changes in vivo and observed in real-time, [8] which is not readily achievable in other commonly used animal models such as mice.

Similar to mammals, the zebrafish pancreas is comprised of two types of glandular tissues, both of which carry out essential physiological functions. [2], [9], [10] The endocrine tissue is critical for the regulation of glucose metabolism through secretion of insulin, somatostatin, and glucagon directly into the bloodstream. Zebrafish genes coding insulin and glucagon as well as other important proteins in the regulation of glucose metabolism function with similar regulatory patterns and activities as seen in mammalian counterparts. [11].

While there is no suitable animal models that could recapitulate human diabetic vasculopathy available, [2] zebrafish becomes an attractive target animal for the development of novel small animal models of diabetic vascular disease. Indeed, immersing zebrafish in glucose solution or destroying the pancreas by streptozotocin can elevate organ glucose concentrations from 50–75 mg/dl to approximately 300–400 mg/dl. [11], [12] This acute hyperglycemia impairs glomerular basement membrane, photoreceptor layer, inner plexiform layer and inner nuclear layer in adult zebrafish. It is further shown that larvae with hyperglycemia displayed altered cardiac development, [13] insulin and PEPCK expression. [14] Olsen et al. found that the fin regeneration was impaired in STZ induced hyperglycemia adult zebrafish. [12].

A high cholesterol diet induced vascular lipid accumulation and inflammation in zebrafish larvae have also been proposed as a model for real-time study of early atherogenesis in vivo. [15] With this high cholesterol diet, Fang et al. characterized the oxidized lipid milieu in HCD-fed zebrafish larvae, and further conformed the HCD-fed zebrafish as a valuable model to study early processes of atherogenesis. [16] However, a zebrafish model of type 2 diabetes with vascular complications remains to be established.

In the present study, we developed a combination of high cholesterol diet and high glucose environment to induce vascular abnormalities in zebrafish larvae that are characterized with metabolic disturbances similar to type 2 diabetes. We also established methodologies for the quantification of some metabolic parameters and the tracking of vascular abnormalities in living larvae.

## Methods

### 1: Zebrafish

Zebrafish maintenance and procedures were conducted in accordance with National Institutes of Health guidelines of the use and care of experimental animals and approved by the Institute Animal User and Ethical Committees at Shandong University. High cholesterol diet (HCD) was made by soaking egg yolk in a diethyl ether solution of cholesterol (Solarbio) to achieve a content of 10% (w/w) cholesterol in the food after ether evaporation. To determine the location and amount of vascular lipid accumulation in larvae, both control and HCD food were supplemented with 10 µg/g of a fluorescent cholesteryl ester analog, cholesteryl BODIPY®558/568 dodecanoic acid (Invitrogen). Detailed method can be found in Supporting Information S1.

### 2: Biochemistry analysis

Thirty larvae of each experimental group were euthanized, and washed twice in double distilled water. Abdomens containing undigested food were removed, and the remaining body parts were pooled and homogenized as described previously.[16] Protein concentrations in the homogenates were determined using BCA assays. Levels of glucose, total cholesterol and triglycerides in homogenates were measured, and the results were standardized by the total protein levels of the homogenates.

### 3: Real-time PCR

Ten larvae were euthanized and homogenized as mentioned above. Thereafter, the tissues were used for isolation of total RNA using TRIzol (Invitrogen) according to the manufacturer's instructions. The isolated RNA was reverse transcribed (Fermantas) and cDNA was stored −20°C. For fluorescent detection of PCR products, reactions containing template and specific primers were amplified with sybergreen (Table 1).

**Table 1:** Primers for RT-qPCR.

Gene	Forward Primer	Reverse Primer
β-actin	CATCAGGGTGTCATGGTTGGT	TCTCTTGCTCTGAGCCTCATCA
Glucagon	AAGCGAGGAGACGATCCAAA	TCCAACACACACCAGCAAATG
Insa	GAGCCCCTTCTGGGTTTCC	AAGTCAGCCACCTCAGTTTCCT
Pck1#	CATCACGCATCGCTAAAGAG	GCTCTCAGATTCCCTTCTTTGTC

Primers used for real time-PCR. We checked the expression levels of glucagon, insulin, PEPCK. All three genes in HCD-HG group were expressed higher than those in the control group, displaying some characteristics of type 2 diabetes. In zebrafish, there are two insulin isoforms named Insa and Insb. Insa functions similarly to the mammalian homologue in glucose regulation, whereas the Insb plays a more important role in development. Therefore, in our study we take Insa as the index of total insulin.

# Pck1: phosphoenolpyruvate carboxykinase (PEPCK).

### 4: Confocal microscopic analysis in vivo

For in vivo confocal microscopy, anaesthetized zebrafish larvae were fixed in a cell culture dish (NEST) with glass bottom in low melt point agrose (Solarbio) as described previously.[3] A Nikon eclipse Ti and UltraVIEW®VOX confocal microscope was used to exquisite information in either regular or spectral acquisition modes (details can be found in Supporting Information S1).

### 5: Statistical analysis

Data were expressed as means ±SD and values were compared using Student's t test with two-tailed p<0.05 being considered statistically significant.

## Results

### 1: Combined high cholesterol diet and 3% glucose solution immersing induces a rapid onset of diabetes in zebrafish larvae

Considering hyperglycemia and hyperlipidemia frequently co-exist in human type 2 diabetes,[17] we postulated that an adequate exposure of the combined high glucose and high cholesterol diet may induce metabolic disturbances that are similar to diabetes. Hence, the vascular changes in these zebrafish may reflect the similar changes observed in established mammalian diabetic models. As zebrafish larvae body is transparent during approximately 30 days post-fertilization (dpf), [3] we developed the zebrafish larvae model of diabetic vasculopathy, which could occur in a relative short time period and the vascular changes can be quantified in living zebrafish.

In our pilot studies, we applied wild type zebrafish larvae with different concentrations of glucose (1%∼5%) and high cholesterol diet (10% cholesterol, about 3 mg/larva, twice a day, each for 2 hours). Using a fluorescent cholesteryl BODIPY®558/568 dodecanoic acid, which enables microscopic tracking in vivo, we explored the optimal glucose concentrations and feeding time periods that could result in lipid accumulation in the vasculature of larvae as analyzed by real-time confocal microscopy. We showed that it took more than 3 weeks to induce lipid accumulation in the vascular wall with glucose concentrations of 1% or 2%, whereas glucose concentrations of 4% or 5% caused a high mortality (>75%) within 10 days (data not shown). At a concentration of 3% glucose, a high cholesterol diet induced a clear lipid deposit in the vasculature within 10 days and ∼90% of the larvae survived for more than 15 days during the exposure.

**Figure 1:**
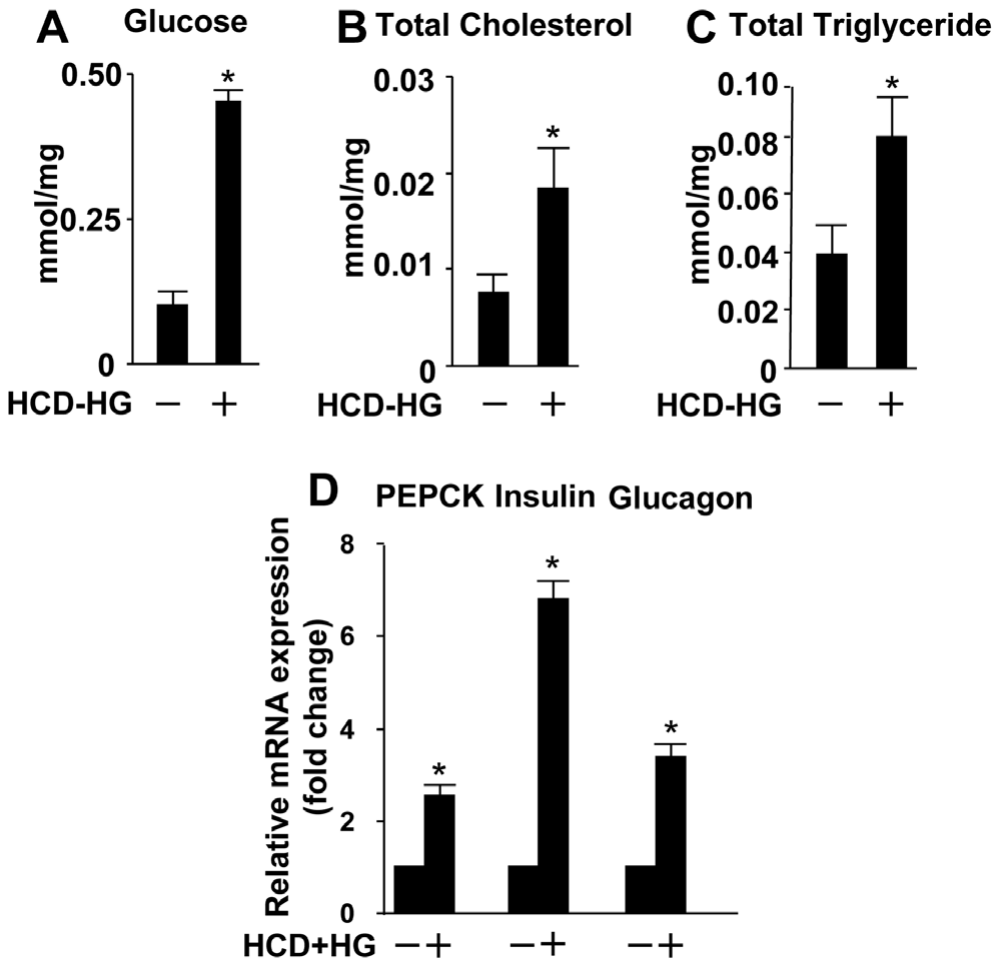
Characteristic biochemical profiles of Type 2 diabetes in zebrafish larvae. The results are presented as mean ±SD. After 10 days of the HCD-HG treatment, the zebrafish displayed increased levels of glucose (A), total cholesterol (B), and total triglycerides (C). The mRNA levels of insulin, glucagon and PEPCK also increased (D). For the measurements of these biomarkers, 30 larvae were homogenized in each group, and we repeated for 3 times. Levels of glucose, TC, and TG were normalized by the total protein. Asterisk: Comparison of glucose, TC and TG respectively between control group and HCD-HG group, p<0.05.

To determine whether the formation of vascular lesion is linked to the onset of diabetes under this experimental condition, we examined metabolic profile of wild type zebrafish larvae in the control group and the HCD-HG treatment group. Because of the small size of a larva, it is not feasible to directly collect blood samples. Therefore, we measured total cholesterol and triglyceride levels in the whole body lysate as described previously.[16], [18], [19] It should be acknowledged that the relationship in the levels of glucose, cholesterol and triglyceride between whole body lysate and blood remains to be established. Compared to the group fed with control diet, all three parameters were increased by several folds in the group fed the HCD-HG (Fig. 1A–C).

In addition, since there are no special antibodies or other reagents that target the insulin and glucagon in zebrafish larvae, we examined the effect of HCD-HG on the corresponding gene expression levels by real-time quantitative PCR. Due to the small body size of the zebrafish larvae, it is practically not possible to isolate specific tissues for the tissue specific mRNA quantifications without noticeable contaminations from surrounding tissues. Therefore, we examined the effect of HCD-HG on the corresponding gene expression levels by real-time quantitative PCR using the mRNAs extracted from the whole body lysate. As the expression level of phosphoenolpyruvate carboxykinase (PEPCK), a critical enzyme that catalyzes the conversion of oxaloacetate to phosphoenol pyruvate, is negatively correlated with the level of blood glucose in acute hyperglycemia while up-regulated by chronic hyperglycemia in type 2 diabetes, [20], [21] we also determined the effect of HCD-HG treatment on the expression of PEPCK by real-time PCR. As shown in the Figure 1D, the HCD-HG treatment for 10 days dramatically up-regulated the expression of insulin, glucagon and PEPCK in zebrafish larvae. Taken together, these results suggest that HCD-HG treatment for 10 days induced diabetic status in zebrafish larvae.

### 2: Combined high cholesterol diet and 3% glucose solution immersing induced vascular lesion in zebrafish larvae

We then studied whether the HCD-HG treatment-induced diabetes is associated with the development of vascular abnormalities in zebrafish larvae. We applied real-time confocal microscopic analysis of endothelial cell damage, vascular lipid accumulation and inflammatory cell infiltration, and blood flow velocity in transgenic zebrafish larvae in which endothelial cells, myeloid cells, and red blood cells are genetically labeled with fluorescent proteins. Moreover, we tested the therapeutic efficacy of thiazolidinedione (TZD) pioglitazone and biguanide metformin, well-established anti-diabetic drugs, [11], [15] on the potential vascular complications in this model.

#### 2.1: Endothelial layer thickness

**Figure 2:**
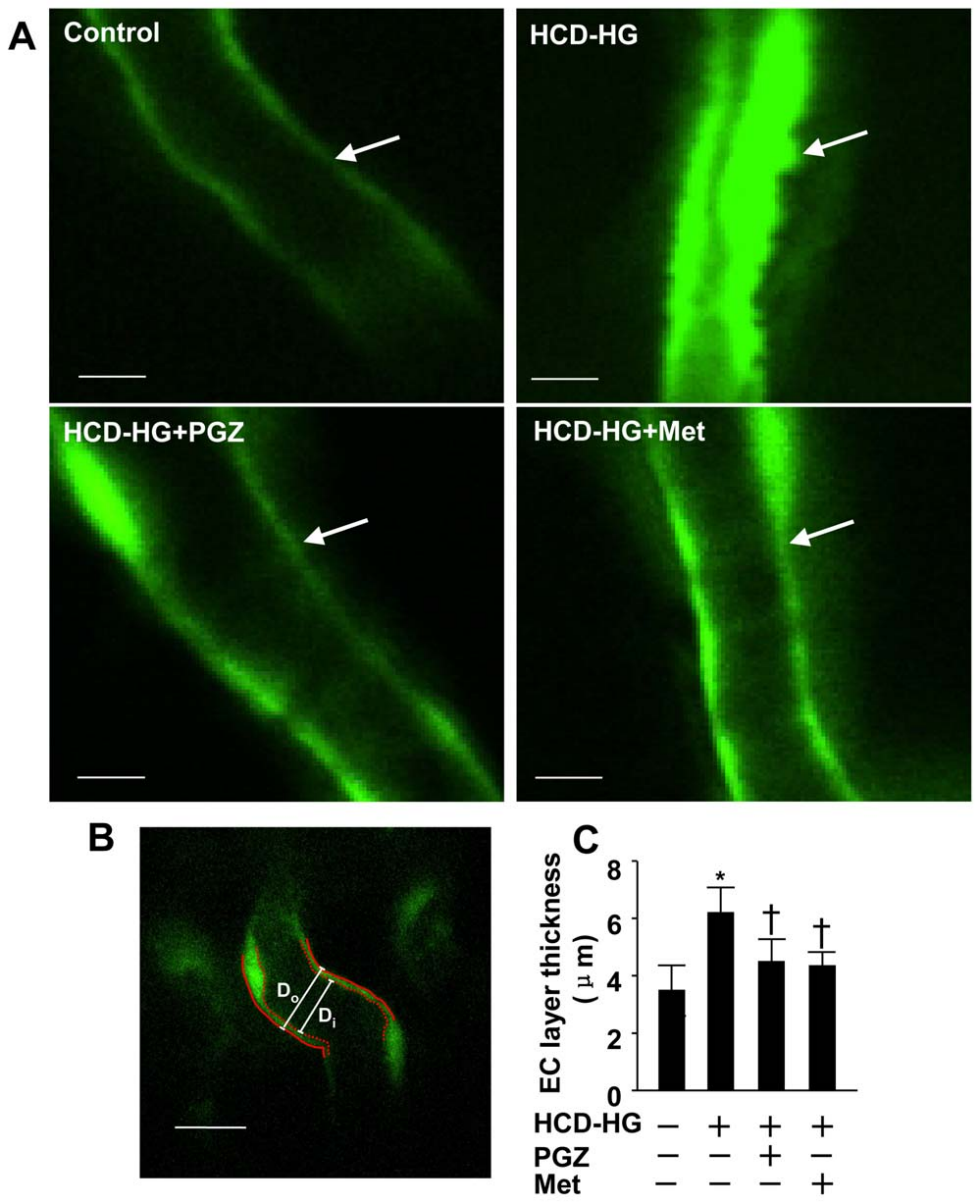
Endothelial layer thickening of optical vessels in HCD-HG treatment group. A: Different treatments led to changes of endothelial layer in optical arteries. Transgenic zebrafish (Fli1:EGFP) larvae were imaged in a lateral position by confocal microscope, and emission wavelength of 488 nm was detected. Scale bar  = 10 µm. B: Endothelial layer thickness (T) was measured by subtracting inner diameter (Di) from outer diameter (Do), T = (Do-Di)/2. Changes in the thickness of endothelial layer were quantified and presented as mean ±SD. Scale bar  = 20 µm. C: Pioglitazone and metformin effectively prevented the endothelial layer from becoming thick afer HCD-HG treatment. N = 11 in the control group, N = 24 in the HCD-HG group, N = 10 in HCD-HG+ pioglitazone group, N = 18 in HCD-HG+ metformin group. PGZ: pioglitazone; Met: metformin. Asterisk (*): Comparison of EC layer thickness between control group and HCD-HG group (p<0.05). Cross (†): Comparison of EC layer thickness between control and drugs treatment groups (p<0.05).

In mammalians, the damage of endothelial cells, especially retinal capillary endothelium, is closely linked with hyperglycemia in a setting of diabetes. In zebrafish, several studies have established that hyperglycemia and hyperlipidemia can cause endothelial damage. [15] Therefore, we examined whether our diabetic model recapitulates the endothelial damage. Confocal microscopic analysis showed that 10-day exposure of the HCD-HG caused a demonstrable thickening of the endothelial layer in optic artery in *fli1:EGFP* larvae (Figure 2A). We quantified the thickness of optic artery endothelial layer by subtracting inner diameter from outer diameter (Figure 2B), and confirmed the presence of endothelial thickening in the group fed the HCD-HG. We further observed that this vascular lesion was significantly inhibited by the treatment of pioglitazone or metformin (Figure 2C).

#### 2.2: Lipid and myeloid cell accumulation in vasculature

**Figure 3:**
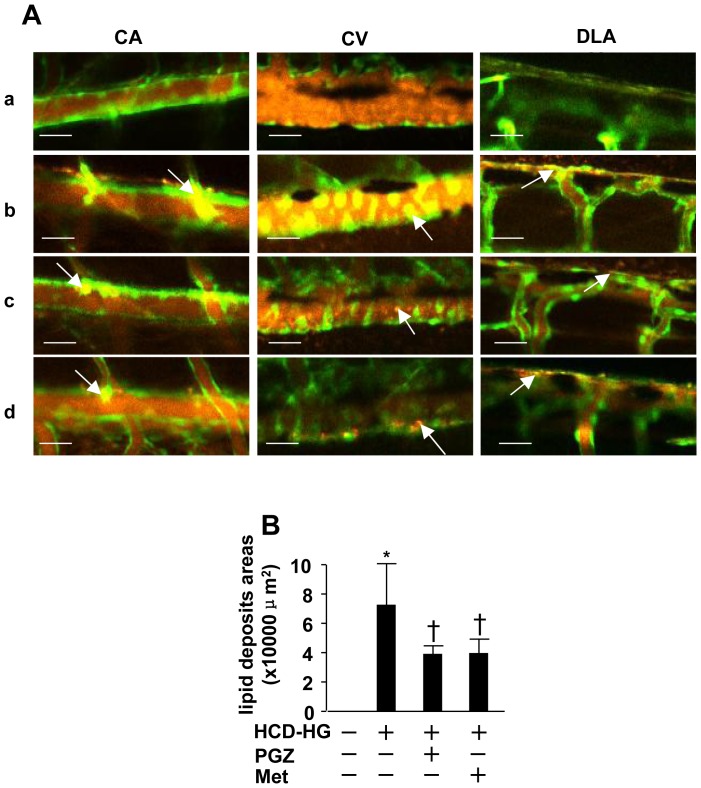
Lipid accumulation in vasculature. A: Apparent lipid accumulation (yellow) was observed. (a), (b), (c), (d) panels respectively show lipid deposits in different experimental groups. (a) Control group; (b) HCD-HG group; (c) Pioglitazone treatment group; (d) Metformin treatment group. White arrows point out the locations of lipid deposits. Scale bar  = 40 µm. B: Areas of the lipid accumulation were selected as the region of interest (ROI), and estimated by measuring the dimension of ROI. Using the Student t test, we found that the amount of lipid accumulation was significantly larger in the HCD-HG treated group than that in the control group (p<0.05). The addition of pioglitazone or metformin significantly reduced the lipid accumulation (p<0.05). Asterisk (*): Comparison of lipid accumulation between control group and HCD-HG group. Cross (†): Comparison of lipid accumulation between HCD-HG group and drug treatment groups, respectively.

Using *fli1:EGFP* and Lyz:EGFP larvae, we further analyzed lipid and myeloid accumulation as previously reported. [15] Feeding *fli:EGFP* with a control diet and immersing in pure water or a high cholesterol diet enriched with cholesterol BODIPY®558/568 cholesteryl ester (orange) and at the same time immersing in the 3% glucose solution for 10 days resulted in the appearance of diffused orange fluorescence in the vasculature, consistent with circulating fluorescent lipid. Furthermore, the HCD-HG treatment led to accumulation of bright orange fluorescence in focal areas of blood vessels including the dorsal longitudinal anastomotic vessel (DLAV), caudal aorta (CA) and the caudal vein (CV) (Figure 3A), suggesting vascular lipid accumulation. As the majority of lipid deposits were observed in the DLAV and CA, we measured the area of the lipid accumulation in DLAV and CA in different experimental groups. As shown in Figure 3B, HCD-HG induced a significant lipid accumulation in the vessels, which was suppressed by the treatment of pioglitazone or metformin.

**Figure 4:**
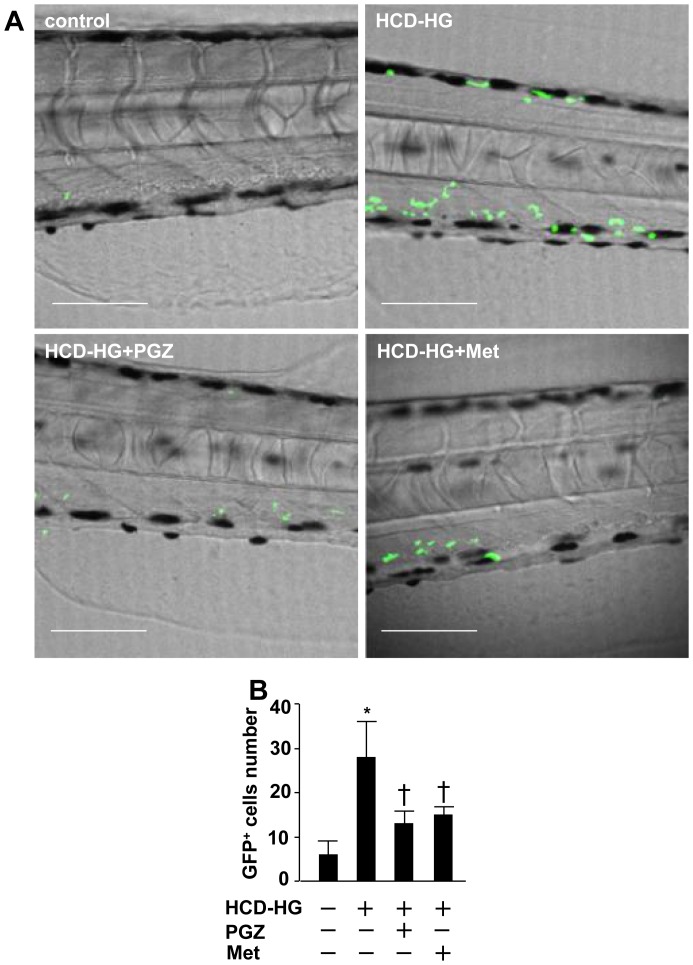
HCD-HG treatment induced inflammatory cells infiltration. A: HCD-HG treatment resulted in the increased number of GFP^+^ cells around the caudal vein. B: GFP^+^ cells that locate within 50 µm from the lumen of the caudal artery were counted. n>5 in each group and the experiment was repeated for three times. The results were compared between normal and HCD-HG treated group, HCD-HG treated group and HCD-HG+ pioglitazone or metformin group respectively compared between the four groups by Student t test, p<0.05. Scale bar  = 80 µm. Asterisk (*): Comparison of the number of GFP^+^ cells between control group and HCD-HG group. Cross(†): Comparison of the number of GFP^+^ cells between HCD-HG group and drug treatment groups, respectively.

Using *lyz:EGFP* larvae, in which EGFP is constitutively expressed in granulotytes/microphages, we tracked the recruitment of GFP^+^ myeloid cells to the caudal veins within 2-cell distance from the lumen for each experimental group. While only occasional GFP^+^ cells was observed in the vasculature of larvae fed control diet, there was a robust increase in GFP^+^ cell recruitment in the HCD-HG fed larvae (Figure 4A), suggesting that HCD-HG induces vascular inflammation. The HCD-HG-induced vascular inflammatory response, however, was inhibited by pioglitazone or metformin (Figure 4B).

In summary, our data demonstrated that HCD-HG treatment for 10 days induced vascular abnormalities in zebrafish larvae. In addition, compared to the vascular injuries induced by a high cholesterol diet alone, [22], [23] the vascular abnormalities were exaggerated by the combination of high cholesterol diet and 3% glucose solution immersing, suggesting the possibility of diabetic vasculopathy in our model.

### 3: Combined high cholesterol diet and 3% glucose solution immersing decreased blood flow

In Type 2 diabetic patients, the abnormality in glucose and lipid metabolism leads to red blood cells aggregation, impaired deformability and abnormal platelet adhesiveness, which contribute to a slower blood flow. [24] Thus, we determined whether our model could recapitulate the impaired hemodynamics of blood flow.

**Figure 5:**
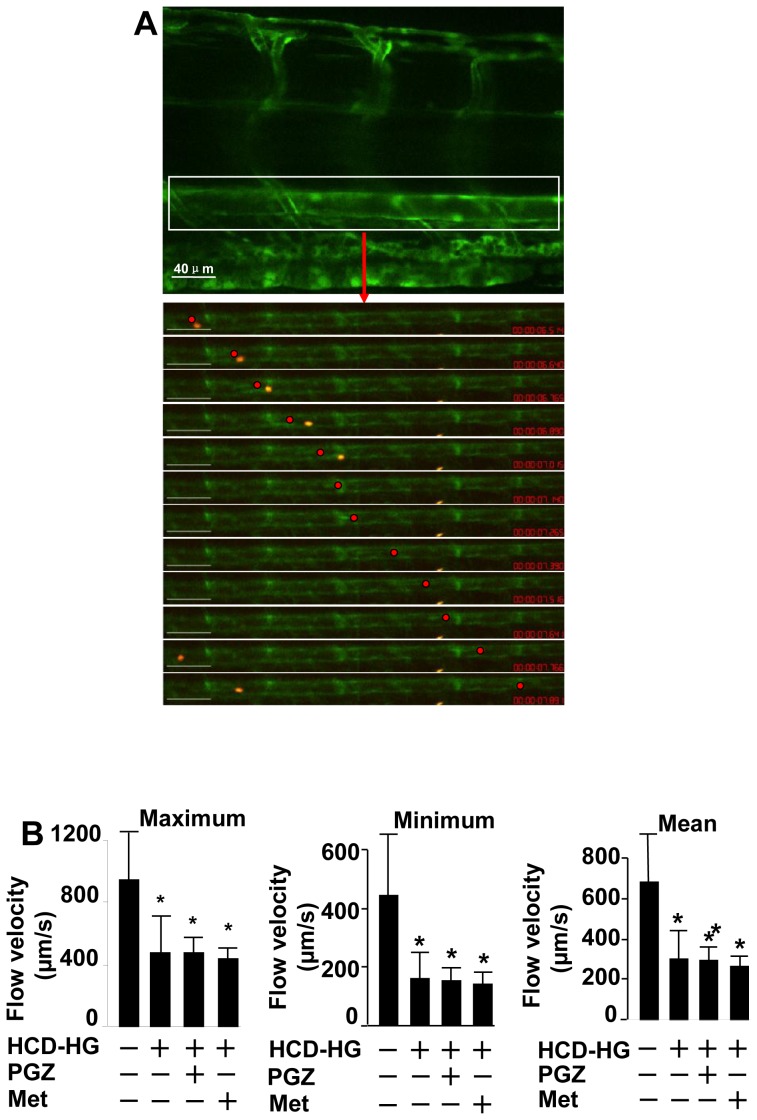
HCD-HG treatment led to reduced velocity of blood flow. A: Velocity of the blood flow in the caudal aorta (CA) was measured. An example of the red blood cell tracking was present in the CA. Each cell was tracked for more than 10 time points, and corresponding frames were produced; motion trajectory was estimated. Relative time and distance were used for the calculation of the velocity. Upper panel scale bar:40 µm; the beneath panel scale bar: 80 µm. B: The values of maximum, minimum, and mean velocities were compared between normal and HCD-HG treated group, HCD-HG treated group and HCD-HG+ pioglitazone or metformin group respectively by Student's t test (n>5 in each group). Asterisk (*): Comparison of flow velocity between control group and corresponding groups, p<0.05.

Using double transgenic *gata1:dsRed/fli1:EGFP* larvae, we tracked the movement of red blood cells (red) in the caudal artery for each experimental group. In the control larvae fed with normal diet, we observed that red blood cells moved with different speeds in the caudal arteries: the highest speed of 1576 µm/s, the lowest of 161 µm/s, the majority distributed between 300∼1100 µm/s with an average of 678±242 µm/s, which is similar to those previously reported.[25], [26] In larvae treated with HCD-HG, the blood flow significantly reduced: the highest speed of 475±229 µm/s, the lowest of 161±88 µm/s, and an average of 300±143 µm/s (Figure 5). However, the reduced blood flow velocity could not be restored by the treatment of pioglitazone or metformin. The mechanism for the reduced blood flow is not clear.

## Discussion

Despite numerous hypotheses, mechanisms of diabetic vasculopathy are still not fully understood. It has been widely accepted that prolonged exposure to hyperglycemia, dyslipidemia, inflammation, and coagulopathy could contribute to diabetic micro- and macro-vascular diseases. However, large scale clinical trials have revealed that lowering glucose levels does not always translate to the reduction in cardiovascular events in patients with type 2 diabetes. [27], [28] Hyperlipidemia seems not the only causative contributor to the diabetic vasculopathy; the roles of the other risk factors remain to be established. [27], [28] Progress in understanding the mechanisms are largely impeded by the lack of appropriate animal models. In an effort to search for an appropriate animal model, we tested the novel diabetic zebrafish larvae model to explore the vascular changes in the presence of hypercholesterolemia and hyperglycemia. Our results clearly demonstrated that a combination of high cholesterol diet and high glucose solution immersing induced a rapid onset of diabetic like metabolic changes. In these larvae, significant vascular abnormalities including endothelial cell damage, vascular lipid accumulation and inflammatory cell infiltration, and decreased blood flow velocity following the establishment of the diabetic condition suggests a possible causal relationship. Therefore, our zebrafish model may be a useful animal model for diabetic vascular complications. The model may be particularly informative for pathophysiological changes in early stages of diabetic vascular disease, which is a crucial time window for any effective therapeutic interventions. Due to numerous technological limitations especially the inaccessibility to blood samples or tissue specific isolations in the small fish larvae, validation of the current zebrafish model by comparing to established mammalian diabetic animal models will be needed. However, because zebrafish is a suitable vertebrate species for large-scale screening of biological pathways and drug targets, the potential application in diabetic research, especially in the screening of candidate drug compounds, is the strength.

To develop the zebrafish model that has metabolic disturbances similar to type 2 diabetes, we treated the zebrafish larvae with high cholesterol diet and exposed them to high glucose solution, which caused lipid and glucose metabolic disorder in vivo. The treated larvae displayed several vascular changes that are similar to vascular changes in mammalian diabetic models and human subjects with diabetes. This was accompanied by significantly increased glucose, insulin and glucagon levels and the related gene expressions, all of which indicated possible insulin resistance. The treated zebrafish larvae also showed high level of total cholesterol, which is consistent with the observed vascular pathogenesis in HCD-HG treated zebrafish. Lipid accumulation in vessel wall and surrounding tissues suggest that an early atherosclerotic injury might have occurred. We also found thickening of endothelial layer in both optical vessels and caudal vein; inflammatory myeloid cells infiltrated into the vascular walls; and reduced blood flow. These early vascular changes were attenuated when pioglitazone or metformin were administered at the same time of the HCD-HG treatment. Both drugs partly inhibited the endothelial thickening, inflammatory activation and lipid accumulation in the caudal vein. However, pioglitazone and metformin did not restore the decreased blood flow caused by the HCD-HG.

Like most other animal diabetic models, the current zebrafish model also has limitations. As a lower vertebrate, the pathophysiological conditions of zebrafish differ from those in humans and mammals. The observed vascular changes may also not be manifested with the same pathological presentations as those in larger mammals and human subjects. Not all features in vascular changes as observed in other animal models, such as mice or rabbits, or in human subjects can be attributed to the vascular changes we observed in diabetic zebrafish. Furthermore, zebrafish larvae are too small to acquire accurate biochemical estimations individually as we routinely do in large animals. Their whole bodies needed to be pooled in order to measure these biomarkers. Since there were no antibodies specifically against the zebrafish insulin and glucagon, levels of these proteins could not be quantified in this study.

In the meantime, the zebrafish possess many unique advantages, such as low cost, short lifecycle and large scale drug screening capacity. More specifically, zebrafish can be easily genetically manipulated and tested in vivo longitudinally. Based on these points, the zebrafish diabetic model may be best utilized as a large scale drug screening tool in addition to its applications to the mechanistic research of diabetic vascular complications. However, findings from this model may require further validation in large animal models.

In summary, we believe that the zebrafish model established in this study manifests several biochemical features similar to type 2 diabetes, and is an informative experimental model for investigations of early stage diabetic vascular complications, which is a crucial time window for effective therapeutic interventions. Complemented with availability of large number of genetically manipulated zebrafish, this model can be used to evaluate the role of specific molecular pathways in vascular diseases and therapeutic agents that may specifically regulate the pathways. It can serve as the first line animal model to screen agents that may be potentially developed to treat diabetic cardiovascular diseases.

## Supporting Information

Supporting Information S1 **Details of the experimental methods are provided in the supporting information.**
(DOC)
