# Supplementary material for: Impact of a Combined High Cholesterol Diet and High Glucose Environment on Vasculature
Source: PLoS One. 2013 Dec 9;8(12):e81485. doi: 10.1371/journal.pone.0081485 (PMC3857185; doi:10.1371/journal.pone.0081485)
Supplement: Supporting Information S1 — Details of the experimental methods are provided in the supporting information. (DOC) [file pone.0081485.s001.doc]

**Methods:**

**Zebrafish**

All zebrafish were maintained in a temperature (28°C) and pH (7.0-7.4) controlled multi-tank re-circulating water system (ESEN, EnvironScience) under a 14 h light:10 h darkness cycle ^1^. All experiments with zebrafish were conducted in accordance with National Institutes of Health guidelines of the use and care of experimental animals and approved by the Institute Animal User and Ethical Committees at Shandong University. Wide type zebrafish (AB) were given as presents from Institute of Zoology, Chinese Academy of Science. The lyz:EGFP transgenic zebrafish were granted by Shanghai Institute of Hematology. Transgenic zebrafish lines of *fli1:EGFP*, and *gata1:dsRed* were purchased from Institute of Biochemistry and Cell Biology, SIBS, CAS. Six groups of sexually mature zebrafish (2 females: 4 males per group at the age of approximately six months) were used as broodstock. Embryo fertilized from spontaneous spawning were collected and pooled together in the morning, within 2 h of laying and were distributed into 10 cm diameter sterile Petri-dishes filled with embryo medium for 5 days (10 embryo per Petri dish). After this period, larvae were transferred into 250 ml beakers at 30 larvae each . A double transgenic zebrafish line of *gata1:dsRed/fil1:EGFP* was generated by out-crossing of gata1:dsRed and fil1:EGFP. Taken advantages that myeloid cells such as granulocytes and macrophages express GFP in *lyz:EGFP* , endothelial cells express GFP in *fli1:EGFP* , and red blood cells express dsRed proteins in *gata1:dsRed* , the genotypes of the transgenic zebrafish line was verified by confocal microscopic analysis.

**Diet and treatment**

Egg yolk was chosen as the normal diet for the purpose of making a subsequent high cholesterol diet. High cholesterol diet (HCD) was made by soaking egg yolk in a diethyl ether solution of cholesterol (Solarbio) to achieve a content of 10% (w/w) cholesterol in the food after ether evaporation. To determine vascular lipid accumulation in larvae, both control and HCD food were supplemented with 10 µg/g of a fluorescent cholesteryl ester analog, cholesteryl BODIPY®558/568 dodecanoic acid (Invitrogen) .

At the 5^th^ day post-fertilization (dpf), larvae of AB and transgenic lines were fed with a normal diet or HCD and high glucose (HG, 3% glucose in nursery water) . HCD-HG treatment kept for 10 days. In addition, some larvae fed with HCD-HG were further treated with pioglitazone or metformin that were mixed with HCD food at a proportion of 1.5 mg/g HCD. All larvae were fed twice a day with approximate amount of 3 mg per larva.

**Biochemistry analysis**

Thirty larvae of each experimental group were euthanized by prolonged exposure to tricaine, and then washed in double distilled water twice. Abdomens containing undigested food were removed, and the remaining body parts were pooled and homogenized in 200 µl of ice-cold PBS in an Eppendorf tube using a plastic pestle . Protein concentrations in the homogenates were determined using BCA assays. Glucose levels in homogenates were measured by ONETOUCH UltraVue ( Johnson&Johnson). The glucose levels were standardized by the total proteins of the homogenates. Total cholesterol in zebrafish larvae homogenates were measured using automated Cholesterol Gen. 2 (CHOL2, Roche, Cat. No. 04718917190), and total triglycerides were measured by triglycerides (TRIGL, Roche). Total cholesterol and triglycerides were both standardized by the total protein levels of the homogenates.

**Confocal microscopic analysis in vivo**

For *in vivo* confocal microscopy, anaesthetized zebrafish larvae were fixed in a cell culture dish with glass bottom (NEST) by low melt point agrose (Solarbio) in 0.003% tricaine nursery water . A Nikon eclipse Ti and UltraVIEW®VOX confocal microscope was used to exquisite information in either regular or spectral acquisition modes. In our study, fluorescence of endothelial cells (green), myeloid cells (green), erythrocytes (red), and lipid accumulation (orange) were respectively detected in corresponding channels. Images were analyzed using software Volocity (PerkinElmer).

**Endothelial layer thickness**

Endothelial layer thickness of *fli1:EGFP* larvae optical artery was imaged in a lateral position. We quantified the thickness (T) using the formula: T= (Do-Di)/2 (Figure 2B). The optic arteries of 30 *fli:EGFP* larvae for each experimental group were analyzed.

**Lipid and myeloid cell accumulation in vasculature**

Lipid accumulation of *fli1:EGFP* larvae fed with the fluorescent cholesteryl BODIPY®558/568 dodecanoic acid was imaged in a lateral position. The dorsal longitudinal anastomotic vessel, caudal aorta and caudal vein in trunk/tail area were imaged with 20X objective. Fluorescent sedimentations detected in 561nm were considered as lipid accumulation (orange), and quantified by area. In a lateral position, the infiltration of inflammatory cells (green) around the caudal vein in *lyz:EGFP* larvae were imaged. GFP positive cells within 50 µm from the lumen and along 600 µm of the caudal artery in 5 *lyz:EGFP* larvae for each experimental group were counted.

**blood flow velocity**

The velocity of blood flow in the caudal artery of *gata1:dsRed/fli1:EGFP* larvae was measured. Briefly, the larvae were fixed laterally on a piece of 0.17 μm thickness cover glass, and about 600 μm length was imaged by reading at maximal speed and short exposure time (76 ms for GFP and 4 ms for RFP). Each larva was observed for 15s; 117 images were collected. The positions of individual RFP positive cells (red) in the series images as well as the relative time for the imaging and moving distance of each individual cell were recorded (Figure 5A) for calculating the cell’s velocity at different time points using a formula of cell’s velocity = moving distance/ relative time. The cells we took in each image were distinguishable. We recorded the tracks of these cells, and then there was a chart automatically generated, which recorded the detailed information of those cells (software Volocity, PerkinElmer ). Only cells that stayed in focus were tracked at least along a distance of 250 μm for the analysis (total distance of collection was 600 μm). Red blood cells, which did not move or moved into the segmental vessels, were excluded from the final analysis. We were thereby able to obtain instantaneous flow velocity profiles for individual erythrocytes over a 2-4 second period of measurement. The mean, maximum and minimum velocities of all measured cells (3~5 cells per larvae and 10 larvae for each group) were calculated.

**Statistics**

Data were expressed as means ± SD and values compared using Student's t test. An overall value of p＜0.05 was considered statistically significant.

Reference:

1. M.Westerfield (1995) The Zebrafish Book.

2. Hall C, Flores MV, Storm T, Crosier K, Crosier P (2007) The zebrafish lysozyme C promoter drives myeloid-specific expression in transgenic fish. BMC Dev Biol 7: 42.

3. Lawson ND, Weinstein BM (2002) In vivo imaging of embryonic vascular development using transgenic zebrafish. Dev Biol 248: 307-318.

4. Traver D, Paw BH, Poss KD, Penberthy WT, Lin S, et al. (2003) Transplantation and in vivo imaging of multilineage engraftment in zebrafish bloodless mutants. Nat Immunol 4: 1238-1246.

5. Stoletov K, Fang L, Choi SH, Hartvigsen K, Hansen LF, et al. (2009) Vascular lipid accumulation, lipoprotein oxidation, and macrophage lipid uptake in hypercholesterolemic zebrafish. Circ Res 104: 952-960.

6. Gleeson M, Connaughton V, Arneson LS (2007) Induction of hyperglycaemia in zebrafish (Danio rerio) leads to morphological changes in the retina. Acta Diabetol 44: 157-163.

7. Fang L, Harkewicz R, Hartvigsen K, Wiesner P, Choi SH, et al. (2010) Oxidized cholesteryl esters and phospholipids in zebrafish larvae fed a high cholesterol diet: macrophage binding and activation. J Biol Chem 285: 32343-32351.
